# Coverage and effectiveness of intermittent preventive treatment in pregnancy with sulfadoxine–pyrimethamine (IPTp-SP) on adverse pregnancy outcomes in the Mount Cameroon area, South West Cameroon

**DOI:** 10.1186/s12936-020-03155-2

**Published:** 2020-03-02

**Authors:** Judith K. Anchang-Kimbi, Laken N. Kalaji, Harry F. Mbacham, Godlove B. Wepnje, Tobias O. Apinjoh, Irene U. Ngole Sumbele, Jodie Dionne-Odom, Alan T. N. Tita, Eric A. Achidi

**Affiliations:** 1 Department of Zoology and Animal Physiology, Faculty of Science, University of Buea, P.O. Box 63, Buea, Cameroon; 2 Department of Biochemistry and Molecular Biology, Faculty of Science, University of Buea, P.O. Box 63, Buea, Cameroon; 3 Department of Medicine, University of Alabama at Birmingham, Birmingham, USA; 4 Department of Obstetrics and Gynecology, University of Alabama at Birmingham, Birmingham, USA

**Keywords:** Birth weight outcome, Cameroon, IPTp-SP (doses) coverage, Placental malaria infection, Pregnancy

## Abstract

**Background:**

Growing concerns about the waning efficacy of IPTp-SP warrants continuous monitoring and evaluation. This study determined coverage of IPTp-SP and compared the effectiveness of the 3-dose to 2-dose regimen on placental malaria (PM) infection and low birth weight (LBW) in the Mount Cameroon area.

**Methods:**

Consenting pregnant women were enrolled consecutively through a cross-sectional survey at delivery at four antenatal clinics, two each from semi-rural and semi-urban settings from November 2016 to December 2017. Reported IPTp-SP use, demographic and antenatal clinic (ANC) data of the mothers and neonate birth weights were documented. Maternal haemoglobin concentration was measured using a haemoglobinometer and PM infection diagnosed by placental blood microscopy. Logistic regression analysis was used to model study outcomes.

**Results:**

Among the 465 parturient women enrolled, 47.0% (203), 34.7% (150), 18.3% (79) and 7.1% (33) reported uptake of ≥ 3, 2.1 dose(s) and no SP, respectively. Uptake of ≥ 3 doses varied significantly (p < 0.001) according to type of medical facility, timing of ANC initiation and number of ANC visits. The prevalence of PM was 18.5% where uptake of ≥ 3 SP doses (AOR = 2.36: 95% CI  1.41–4.87), primiparity (AOR = 2.13: 95% CI  1.19–3.81), semi-rural setting (AOR = 1.85: 95% CI  1.12–3.04) increased odds of infection. Also, three or more dosing was associated (p < 0.001) with increased PM density notably among women from semi-urban areas. Compared with third trimester, ANC initiation in the second trimester (AOR: 0.39: 95% CI  0.20–0.74) lower odds of infection. The prevalence of LBW infants was 7.3% and were generally those of anaemic (AOR: 4.6: 95% CI  1.03–20.57) and semi-rural (AOR: 5.29: 95% CI  1.73–16.15) women. Although ≥ 3 (AOR: 0.31: 95% CI  0.11–0.87) and 2 (AOR: 0.32: 95% CI  0.11–0.93) doses of SP was associated with lower odds of LBW, ≥ 3 doses were not associated with additional increase in birth weight nor maternal haemoglobin levels when compared with 2 doses.

**Conclusion:**

In the Mount Cameroon area, reported uptake of IPTp with ≥ 3 SP doses did not provide observable prophylactic benefits. SP resistance efficacy studies are necessary.

## Background

In sub-Saharan Africa, *Plasmodium falciparum* infection increases the risk of maternal anaemia and low birthweight, and prematurity in women and their neonates, respectively [1, 2]. It was estimated that without pregnancy-specific interventions, 9·5 million pregnant women were exposed to infection in 2010, leading to 750,000 low birthweight deliveries. Recommended malaria prophylactic treatment was estimated to prevent 128,000 low birthweight deliveries by 2015 [3]. Despite recent decline in malaria transmission in Africa, the burden of malaria in pregnancy (MiP) in the absence of adequate prevention remains substantial [3]. In geographical areas with high transmission, the impact of malaria on maternal health depends on age, gravidity, trimester of pregnancy, coinfection [1, 4] nutritional status [5] the intensity of malaria transmission (increased in rural populations), season, and use of malaria prevention [6].

In most African countries, SP remains the recommended drug for IPTp. SP provides intermittent clearance or suppression of existing asymptomatic infections from the placenta (the treatment effect) and may prevent new infections from occurring for several weeks by maintenance of suppressive drug levels (the post-treatment prophylactic effect) [7]. Based on the findings of multi-centre trials on the evaluation of IPTp-SP, three or more doses of IPTp-SP were associated with less placental malaria, higher mean birth weight and fewer LBW births compared with two doses [8]. Thus, the WHO policy on IPTp-SP was revised and recommends uptake of at least three SP doses with each dose administered at each scheduled antenatal care visit, at least 1 month apart up to the time of delivery and ideally by direct observed therapy [9]. Although there has been a fivefold increase in the percentage of women receiving the recommended three or more doses of IPTp in 20 African countries, coverage in Cameroon remains low (29%) [10].

The effectiveness of IPTp-SP is threatened by rising levels of parasite resistance to SP in several countries across Africa [11]. Emergence and successive acquisition of polymorphisms in both the *P. falciparum* dihydrofolate reductase/dihydropteroate synthetase (*pfdhfr/pfdhps*) genes are associated with a high-level of SP resistance and clinical treatment failure in several epidemiological settings. Parasites harbouring the K540E mutation, which is a proxy for the *pfdhfr*–*pfdhps* quintuple mutant is strongly linked with resistance rendering SP ineffective to clear *P. falciparum* infections [12]. Sextuple mutant parasites (quintuple mutant plus the additional *pfdhps* A581G mutation) defined as super-resistant parasites [13] have been associated with a loss of IPTp-SP efficacy [14–18]. Although resistance to SP reduces the parasitological efficacy of IPTp, beneficial associations between the use of IPTp-SP and low birthweight are still seen in areas where prevalence of parasites with quintuple mutations is > 90% [11, 12, 17]. IPTp-SP appears to protect against malaria as well as other non-malarial causes of LBW such as sexually transmitted and reproductive tract infections (STIs/RTIs). SP may exert its protection through antibacterial or anti-inflammatory actions [19, 20].

The wide spread of SP super-resistance in Africa is expected with continued SP use and may undermined anti-malarial policies [17]. In Yaoundé, Cameroon, highly resistant parasites (octuple mutation genes) to SP have been isolated from pregnant women with symptomatic *P. falciparum* infection [21]. Also, a high prevalence of *P. falciparum* SP resistant parasites among pregnant [22] and non-pregnant [23, 24] populations has been reported along the slope of Mount Cameroon. On this basis, continuous monitoring and evaluation of SP efficacy in Cameroon is crucial. Thus, this study determined the coverage of IPTp-SP since the transition from a 2-dose to 3-dose IPTp-SP policy in Cameroon in 2012. Secondly, the study ascertained whether 3 or more doses of SP to the 2-dose regimen for intermittent preventive therapy during pregnancy provide any additional benefits in reducing the risk of PM infection and LBW in some selected semi-rural and semi-urban settings in Mount Cameroon area, South West Region, Cameroon.

## Methods

### Ethics statement

The study was approved by the Institutional Review Board of the University of Buea (Ref No: 2016/0351/UB/FHS/IRB), whereas administrative authorization was obtained from the South West Regional Delegation of Public Health. Written informed consent was obtained from all the study participants.

### Study area

The study was conducted at the maternity units in four selected medical facilities (Mutengene Medical Centre (MMC), Bolifamba Health Centre (BHC), Buea, Government integrated health centre Munyenge (MIHC), and Muyuka District hospital (MDH) located on the eastern slope of Mount Cameroon from November 2016 to December 2017. The localities of these antenatal clinics were selected based on the level of malaria transmission intensity; semi-rural (Munyenge and Bolifamba and semi-urban (Muyuka and Mutengene) settings. Munyenge is a semi-rural setting, endemic for urogenital schistosomiasis and located about 27 km from Muyuka, a semi-urban town in the Mount Cameroon area. Muyuka is found at a lower altitude of 87 m above sea level (asl), while that of Munyenge ranges from 87 to 168 m (asl). Both areas have a temperature and average annual rainfall of 24–27 °C and 3126 mm, respectively. These localities are both characterized by a heterogenous population and intensive farming [25]. Mutengene is a semi-urban road-junction town with a highly heterogenous population located at 197 m (asl). Mutengene is characterized by mean temperature of 25 °C, mean relative humidity of 83% (Cameroon Development Corporation (CDC) weather record). Bolifamba is semi-rural community in Buea located at 530 m asl. Buea has a mean relative humidity of 80%, average rainfall of 4000 mm and a temperature range of 18–27 °C [26]. All medical facilities selected for the study are government-owned institutions that offer antenatal care, preventive, curative and delivery services at affordable costs and are highly accessible facilitating utilization of ANC services [27].

In the Mount Cameroon area, malaria parasite transmission is perennial [28]. Pregnant women living in rural areas are highly exposed to malaria parasitaemia (39.2%) [29] than those from semi-urban/urban communities (13.4–22.4%) [30, 31]. Placental malaria in pregnancy in the Mount Cameroon area is most sensitively diagnosed by placental histology compared with placental blood smear microscopy. But placental microscopic infection has epidemiological and clinical significance than low submicroscopic parasitaemia diagnosed by placenta histology only [32]. Cameroon transitioned from a 2-dose to a 3-dose recommendation for IPTp-SP in 2012 [33]. Monitoring studies on the coverage and usage of lPTp-SP and ITN (insecticide-treated nets) in pregnancy in the study area showed that the proportion of women who receive at least two or more doses of IPTp- SP (64%) has increased over the years [30, 34] but levels remain below recommended target (80–100%). Also, recent findings confirm the additional benefits of repeated doses of SP in combination with ITN use in reducing peripheral malaria parasitaemia and maternal anaemia [30].

### Study design and population

Consenting pregnant women were enrolled consecutively through a cross-sectional survey at delivery in health facilities. Enrolment by the time of delivery ensured uptake of adequate doses of IPTp-SP (at least three doses of SP) as stipulated by the World Health Organization (WHO) [35]. Mothers with evidence of complicated pregnancy (including hypertension, preeclampsia, diabetes and twin births) were not eligible for the study. An interview-guided administered questionnaire was used to obtain information relating to demographic data (age, residence) antenatal clinic data (gestational age, parity and number of antenatal care visits), fever history and ITN usage the night prior to the survey. The use of IPTp-SP and number of doses as well as new-born birth weights were documented and verified by checking ANC cards, patient’s medical record book and health centre maternal care register. Axial body temperature was measured using a digital thermometer to determine fever. Evidence of any chronic illness such as HIV/AIDS was noted.

### Sample collection

A maternal finger-prick blood sample was collected before delivery for haemoglobin (Hb) measurement. After the delivery of the placenta, a small piece of tissue (0.5 cm^2^) from a healthy paracentric area (approximately a quarter of the distance from the centre of the placenta) was excised to prepare impression smears for detection of PM infection [32]. All samples were transported on ice bath to the Malaria Research Laboratory, University of Buea for analysis.

### Sample processing and analysis

#### Haematological analysis

Haemoglobin concentration was determined using a haemoglobinometer (HemoCue, Angelholm, Sweden). Maternal anaemia was defined as a haemoglobin level of < 11 g/dL [36]. Anaemia severity was defined as follows: mild anaemia (Hb: 10–10.9 g/dL), moderate anaemia (Hb: 7–9.9 g/dL), and severe anaemia (Hb < 7.0 g/dL) [36].

#### Placental malaria parasitaemia determination

Thin impression smears of placental blood were prepared and stained with 10% Giemsa (Sigma-Aldrich, St. Louis, MO), as described previously [32]. The malaria parasitaemia status (the presence of any malaria parasite stages; trophozoites, schizonts) and density were determined under oil immersion with the 100× objective of a binocular Olympus microscope. Blood smears were considered negative if no parasites and/or pigment in macrophages were found after counting 100 high-power fields. To determine the percentage of malaria parasitization in the placenta, malaria parasite-infected red cells were counted against 1000 erythrocytes. All slides were read by 2 independent microscopists blinded to each other’s results. In case of discordant findings, a third reader was used.

### Outcome variables

The primary study outcomes were placental malaria infection and birth weight. Secondary outcomes included maternal haemoglobin levels and anaemia.

#### Definitions

1. Placental malaria infection was defined as detection by microscopy of *P. falciparum* asexual stages, any density and/or haemozoin in macrophages.
2. Low birth weight was defined as a birth weight < 2500 g.
3. Anaemia was defined as haemoglobin concentration < 11 g/dL.
4. Coverage of IPTp-SP was defined as uptake of SP (doses) by the time of delivery.
5. Febrile status was defined as body temperature greater than 37.5 °C.
6. Placental malaria parasite density was expressed as percentage of parasitization.

### Independent variables

Demographics included setting (semi-rural and semi-urban), maternal age (< 21, 21–25, > 25 years), parity status (primiparae, secundiparae, multiparae) and ITN usage. Semi -rural areas (Munyenge and Bolifamba) are geographic areas located outside semi-urban towns of Muyuka and Buea, respectively.

### Data analysis

All data were entered, validated and analysed using SPSS Statistics 20 (SPSS Inc., Chicago, IL). The significance of differences in proportions were explored using the Pearson’s Chi square test, whereas the differences in group means were assessed using Student t test, analysis of variance (ANOVA). Association analysis of number of SP doses, PM infection and LBW was undertaken by multinominal logistic regression. Maternal age, parity, setting, ANC attendance, ITN usage were included in the model as possible confounders. For multivariate models, the variables were selected based on statistical significance in the univariate models (p < 0.2), explanatory plausibility and collinearity considerations. Percentage change in crude odd ratios of confounders associated with risk of PM and LBW were calculated. Missing data are presented in the tables, but data points were generally complete. A p value < 0.05 was considered statistically significant.

### Sample size determination

The sample size was calculated using the formula n = Z^2^pq/d^2^ [35] where n = the sample size required, z = 1.96: confidence level test statistic at the desired level of significance, p = 15%: proportion of placental blood malaria parasitaemia at delivery [37], q = 1 − p: proportion of malaria parasite negative and d = acceptable error willing to be committed. N = (1.96)^2^ × 0.15 (1 − 0.15)/(0.05)^2^ = 195.9. The minimum estimated sample size calculated per setting was 196. For statistical power, 238 and 227 consented participants from semi-rural and semi-urban areas were enrolled into the study.

## Results

### Characteristics of pregnant women at delivery

A total of 465 pregnant women were enrolled at delivery among whom, 25 were from Bolifamba Health Centre (BHC) 213 from Munyenge Integrated Health Centre (MIHC), 127 from Mutengene Medical Centre (MMC) and 100 from Muyuka District hospital (MDH). In general, 51.2% and 48.8% of the women were from semi-rural and semi-urban towns respectively. Mean maternal age was 25.82 ± 5.50 years (range 15–42). Maternal demographic and antenatal clinic characteristics were comparable between semi-rural and semi-urban areas. Nevertheless, there was variability in the prevalence of fever, maternal anaemia and LBW between the two settings. ITN use the night prior to enrollment was 67.7%. (Table 1). For efficacy analysis, the women were grouped according to the number of IPTp-SP doses received (≤ 1, 2, ≥ 3).

**Table 1:** Association of demographic, ANC clinic and pregnancy outcome characteristics of parturient women from some semi-rural and semi-urban settings in the Mount Cameroon area

Variable	Category	Semi-rural % (n)	Semi-urban % (n)	p-value^$^
Age group	< 21	20.6 (49)	18.1 (41)	0.691
	21–25	31.5 (75)	30.4 (69)	
	> 25	47.9 (114)	50.6 (117)	
Parity	Primiparae	30.3 (72)	29.5 (67)	0.069
	Secundiparae	23.9 (57)	33.0 (75)	
	Multiparae	45.8 (109)	37.4 (85)	
Number of ANC visits	<4 visits	35.7 (85)	42.3 (96)	0.146
Trimester of First ANC	First	8.0 (19)	6.2 (14)	0.402
	Second	74.7 (177)	72.0 (162)	
	Third	17.3 (41)	21.8 (49)	
Dosage frequency of SP	≥ 3 doses	40.8 (97)	46.7 (106)	0.106
	2 doses	31.1 (74)	33.5 (76)	
	≥ 1 dose	28.2 (67)	19.8 (45)	
ITN usage	Yes	66.4 (158)	69.2 (157)	0.522
Febrile status	Febrile	10.1 (24)	1.1 (2)	< 0.001
PM infection	Positive	21.8 (52)	15.0 (34)	0.056
Anaemia status	Anaemic	78.9 (187)	53.3 (121)	< 0.001
Anaemia severity	Mild	25.6 (61)	37.9 (86)	< 0.001
	Moderate	48.7 (116)	14.5 (33)	
	Severe	4.2 (10)	0.9 (2)	
Birth weight status	Low birth weight	12.6 (30)	1.8 (4)	< 0.001
GMPMD^&^	% parasitisation (range)	0.76 (0.1–16.0)	1.94 (0.1–100)	< 0.001
Hb levels (g/dL)	Mean (± SD)	9.8 ± 1.6	10.8 ± 1.1	< 0.001
Birth weight (kg)	Mean (± SD)	3.2 ± 0.7	3.3 ± 0.5	0.142

*ANC* antenatal clinic, *ITN* insecticide-treated nets, *PM* placental malaria infection, *GMPMD* geometric mean placenta malaria parasite density, *Hb* haemoglobin

^$^Values are from Pearson Chi square test (categorical variables) and ANOVA and Student *t* test (continuous variables)

### ANC attendance and uptake of IPTp-SP

A total of 463 (99.4%) women attended antenatal clinic at least once during pregnancy but only 61.9% (288) completed the recommended four or more ANC visits. Most of the women (73.3%, n = 341) registered for the first ANC visit in the second trimester of their pregnancy (Table 1). The mean gestational age at first ANC visit was 22.2 ± 5.9 weeks (range 7–39 weeks). The number of ANC visits made ranged from 1 to 9 with a mean of 3.8 ± 1.3 visits.

**Fig. 1:**
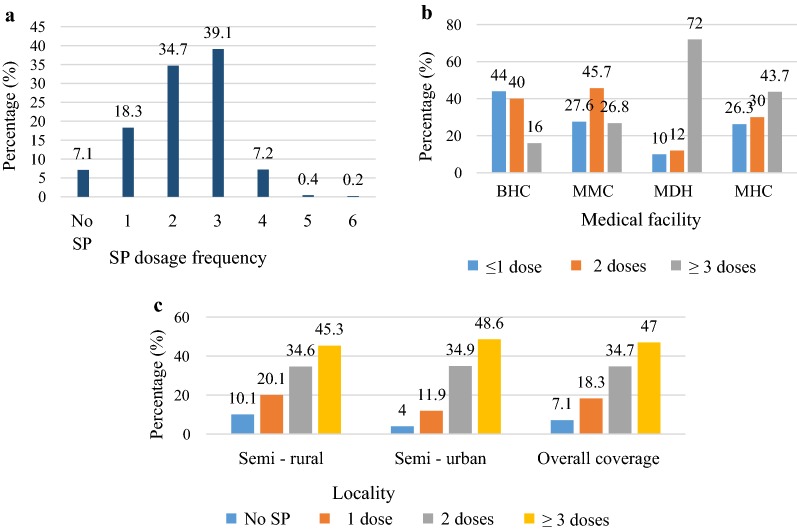
**a** IPTp-SP dose frequency among delivering women. Coverage presented per, **b** medical facility (Bolifamba Health Centre (BHC), Munyenge Intergrated Health Centre (MIHC), Mutengene Medical Centre (MMC) and Muyuka District Hospital (MDC), and **c** setting (semi-rural vs semi-urban)

Thirty-three (7.1%) did not take chemoprophylaxis during their pregnancy giving IPTp-SP coverage of at least one dose of 92.9% (432/465) (range 1–6 doses) which, however was higher (p = 0.010) in semi-urban (96%; 218/227) than in semi-rural setting (89.9%; 214/238). Among the women who took IPTp-SP, the coverage of adequate SP dosage (≥ 3 doses of SP) was 47.0%, (203/432) (95% CI 42.3–52.7) while receipt of partial doses were 34.7% (150) (95% CI 30.4–39.3) and 18.3% (79) (95% CI 14.9–22.2) for 2 doses and 1 dose, respectively (Fig. 1). The prevalence of ≥ 2 doses of IPTp-SP was 81.7% (353/432). Uptake of adequate SP dosage varied significantly (p < 0.001) according to the type of medical facility, the timing of ANC initiation and the number of clinic visits (Table 2). Although IPTp coverage rate of ≥ 3 doses (p = 0.188) was similar between rural and urban areas, the highest uptake was recorded at MDH (72%), followed by MIHC (43.7%), MMC (26.8%) and BHC (16%). The difference was significant (χ^2^ = 58.66; p < 0.001) (Fig. 1). There was association between uptake of ≥ 3 doses of SP and ITN usage (Table 2).

**Table 2:** Association of maternal age, parity status, ANC clinic characteristics and adverse pregnancy outcomes by number of IPTp-SP doses

Variables	Categories	Doses [% (n)]			p-value^$^
		≤ 1	2	≥ 3	
Age group (years)	< 21	24.4 (22)	18.0 (27)	20.2 (41)	0.853
	21–25	28.6 (32)	30.0 (45)	33 (67)	
	> 25	51.8 (58)	52.0 (78)	46.8 (95)	
Parity	Primiparae	27.7 (31)	31.3 (47)	30.0 (61)	0.128
	Secundiparae	20.5 (23)	30.0 (45)	31.5 (64)	
	Multiparae	51.8 (58)	38.7 (58)	38.4 (78)	
Trimester of first ANC visit	First	5.5 (6)	4.7 (7)	9.9 (20)	< 0.001
	Second	53.2 (58)	70.7 (106)	86.2 (175)	
	Third	41.3 (45)	24.7 (37)	3.9 (8)	
Number of ANC visits	< 4 visits	70.5 (79)	45.3 (68)	16.7 (34)	< 0.001
ITN Usage	Yes	58.9 (66)	64.0 (96)	75.4 (153)	0.006
PM infection	Positive	14.3 (16)	17.3 (26)	21.7 (44)	0.245
Anaemia status*	Anaemic	72.2 (70)	63.6 (89)	59.2 (106)	0.102
Birth weight status*	LBW	13.5 (13)	4.3 (6)	4.4 (8)	0.006
GMPMD (range)		^a^1.1 (0.1–63.4) (16)	^b^0.9 (0.1–68) (25)	^c^1.3 (0.1–100) (42)	0.160
Mean Hb levels*		^a^10.0 ± 1.5 (97)	^b^10.5 ± 1.3 (140)	^c^10.5 ± 1.4 (179)	0.015
Mean birth weight*		^a^3.1 ± 0.6 (96)	^b^ 3.3 ± 0.5 (140)	^c^3.4 ± 0.5 (180)	< 0.001

* Analysis excludes 48 women resident in Munyenge and positive for *Schistosoma haematobium* infection

^**$**^Values from Pearson Chi square test (categorical variables) and ANOVA and Post hoc test (continuous variables); *ANC* antenatal clinic, *ITN* insecticide-treated nets, *PM* placental malaria, *GMPMD* geometric mean placenta parasite density, *Hb* haemoglobin

Significant difference between ^b^ vs ^a^: Hb; p = 0.035

Significant difference between ^c^ vs ^a^: Hb; p = 0.011

Significant difference between ^b^ vs ^a^: BWT; p = 0.021

Significant difference between ^c^ vs ^a^: BWT; p < 0.001

No significant difference in Hb and BWT between c and b (p = 1.000)

### Placental malaria infection

A total of 465 placenta biopsies were collected and processed for identification of *P. falciparum* infection. Overall, 18.5% (86) (95% CI 15.2–22.2) women had evidence of PM infection of whom 3.5% (3/86) were diagnosed for haemozoin only. The median (range) placental parasite density was 0.6% (0.1–100%). Generally, placental parasites were scarce: 56.5% (47/83) of actively infected placentas had parasitaemia less than 1% whereas only 17.7% (13/83) showed 10% or more parasitized erythrocytes. Placentas of women from all sites harboured low (< 1.0%) infection levels except those from MDH (7.11%; 0.1–100%) with significantly higher (F = 18.82; p < 0.001) geometric mean parasite density (GMPD) compared with BHC (0.41%; 0.1–1.2%), MMC (0.57%; 0.1–68%) and MIHC (0.81%; 0.1–16.0%).

Placental malaria infection was not reduced in women having taken ≥ 3 doses of IPTp-SP. This observation was evident among primiparae (p = 0.012) where the prevalence of infection was highest among women who received ≥ 3 doses of IPTp-SP than those who had 2 doses or ≤ 1 dose (Additional file 1). More so, uptake of ≥ 3 SP doses was associated with increased odds (AOR = 2.36) of PM infection in multivariate analysis (Table 3). This represents a 42% increased odds of PM infection among women with three or more dosing compared with ≤ 2 dosing (Additional file 2). Other factors associated with higher odds of PM infection include semi-rural setting (AOR = 1.85) and primiparity (AOR = 2.13). ANC initiation in the second trimester (AOR = 0.47) lowered odds of having PM infection (Table 3). Among infected women, placenta parasite density did not differ significantly with uptake of different SP doses (Table 2). However, in semi-urban areas, ≥ 3 SP doses was associated with increased parasite load (p < 0.001) compared with the standard 2-dose regimen. In semi- rural setting, more (≥ 2) doses correlated with reduced parasite density although no additional reduction was seen between ≥ 3 and 2 doses (Additional file 3).

**Table 3:** Risk factors of placental malaria infection

Variable	Category	PM % (n)	Unadjusted OR (95% CI)^a^	Adjusted OR (95% CI)^b^	p-value
Setting	Semi rural (238)	21.8 (52)	1.56 (0.99–2.56)	1.85 (1.12–3.04)	0.016
	Semi urban (227)	15.0 (34)	REF	REF	
Age group (years)^c^	< 21 (90)	27.8 (25)	2.48 (1.37–4.51)	NA	
	21–25 (144)	20.8 (30)	1.70 (0.98–2.95)		
	> 25 (231)	13.4 (31)	REF		
Parity	Primiparae (139)	24.5 (34)	2.09 (1.19–3.68)	2.13 (1.19–3.81)	0.011
	Secundiparae (132)	19.7 (26)	1.59 (0.87–2.88)	1.74 (0.94–3.23)	0.078
	Multiparae (194)	13.4 (26)	REF	REF	
IPTp-SP Dosage frequency	≥ 3 SP dose (203	21.7 (44)	1.66 (0.89–3.10)	2.36 (1.41–4.87)	0.021
	2 SP dose (150)	17.3 (26)	1.26 (0.64–2.48)	1.43 (0.70–2.90)	0.324
	≤ 1 dose (112)	14.3 (16)	REF	REF	
Trimester of first ANC	First (33)	10.5 (9)	0.22 (0.10–0.52)	0.69 (0.25–1.80)	0.448
	Second (339)	16.2 (55)	0.60 (0.34–1.05)	0.39 (0.20–0.74)	0.004
	Third (90)	24.4 (22)	REF	REF	
ITN usage	Yes (315)	19.0 (60)	1.12 (0.68–1.87)	1.02 (0.6–1.73)	0.941
	No (150)	17.3 (17)	REF		

*NA* not applicable, *IPTp-SP* intermittent preventive treatment in pregnancy with sulfadoxine–pyrimethamine, *ANC* antenatal clinic, *ITN* insecticide-treated nets

^a^Values calculated using confidence interval calculator

^b^Values from multinominal regression analysis

^c^Age variable not included in the final model due colinearaity with parity

### Maternal anaemia

The mean maternal Hb levels was significantly different (p = 0.015) according to the number of SP doses taken but not (p = 1.000) between those having received two or three or more doses (Table 2). This observation was similar across parity groups (Additional file 1) as well as the different settings (Additional file 3). In general, ≥ 2 doses (10.5 ± 1.4) was associated with an increase in maternal Hb (mean difference (MD), 0.5 g/dL [95% CI  0.28–0.81 g/dL]; p = 0.002) when compared with < 2 doses (10.0 ± 1.5). Accordingly, anaemia prevalence was highest among women in the < 2 dose group (Table 2) with a significant association seen among multiparous women (Additional file 1).

### Birth weight

Overall, 7.3% (34) (95% CI  5.3–10.1) of newborns were LBW where, most (χ^2^ = 20.04; p < 0.001) of these infants were of mothers living in semi-rural (88.2%; 30/34) than among those from semi-urban (11.8%; 4/34) areas. Semi-rural setting (AOR = 5.82) and maternal anaemia (AOR = 4.6) increased odds of LBW. Three or more doses of SP was not associated with an additional reduction in the occurrence of LBW when compared with the 2-dose regimen (Table 2). This outcome was marked (p = 0.017) in semi-rural setting after stratification analysis (Additional file 2). In multivariate modelling, receipt of 2 (AOR = 0.31) or ≥ 3 (AOR = 0.32) SP doses were less likely associated with odds of delivering a LBW infant than uptake of ≤ 1 dose (Table 4). Adequate dosing was associated with 22% decreased odds of LBW (Additional file 4). Similarly, no difference in mean birth weights was found between the ≥ 3-dose and 2-dose groups (Table 2). Nonetheless, the mean birth weight was significantly higher (p = 0.028) in newborns of primiparae who received ≥ 3 doses of IPTp-SP (3.3 ± 0.5 kg) than those who had ≤ 1 (3.1 ± 0.6 kg), 2 (3.1 ± 0.5 kg) doses after stratifying by parity (Additional file 1).

**Table 4:** Risk factors of low birth weight

Variable	^a^Category	LBW % (n)	Unadjusted OR (95% CI)^b^	Adjusted OR (95% CI)^c^	p-value
Setting	Semi-rural (190)	12.1 (23)	7.62 (2.59–22.52)	5.29 (1.35–16.15)	0.003
	Semi-urban (79)	1.8 (4)	REF	REF	
Age group (years)	< 21 (79)	6.3.(5)	0.6 (0.20–1.86)	0.84 (0.19–3.71)	0.822
	21–25 (128)	10.2 (13)	4.38.(1.79–10.71)	2.22 (0.72–6.89)	0.166
	> 25 (209)	4.3 (9)	REF	REF	
Parity	Primiparae (122)	8.2 (10)	1.46 (0.59–3.63)	1.37 (0.39–4.83)	0.626
	Secundiparae (120)	5.8 (7)	1.0 (0.37–2.70)	1.04 (0.30–3.63)	0.955
	Multiparae (174)	5.8 (10)	REF	REF	
PM infection	Positive (75)	12.0 (9)	2.45 (1.05–5.68)	2.33 (0.90–6.07)	0.083
	Negative (341)	5.3.(18)	REF	REF	
Anaemic status	Anaemic (265)	9.4 (25)	7.71 (1.8–33.02)	4.60 (1.03–20.57)	0.046
	Non-anaemic (150)	1.3 (2)	REF		
IPTp-SP dosage frequency	≥ 3 SP dose (180))	4.4 (8)	0.40 (0.16–1.0)	0.31 (0.11–0.87)	0.027
	2 SP dose (140)	4.3 (6)	0.29 (0.11–0.79)	0.32 (0.11–0.93)	0.036
	≤ 1 dose (96)	13.5 (13)	REF	REF	
ITN usage	Yes (280)	6.1 (17)	1.23 (0.55–2.76)	1.15 (0.46–2.87)	0.762
	No (136)	7.4 (10)	REF	REF	

*PM* placental malaria infection, *IPTp-SP* intermittent preventive treatment in pregnancy with sulfadoxine–pyrimethamine, *ITN* insecticide-treated nets

^a^Counts excludes 48 women resident in Munyenge endemic foci and positive for *Schistosoma haematobium*

^b^Values from confidence interval calculator

^c^Values from multinominal regression analysis

## Discussion

This study reports on the coverage of SP (doses) in the Mount Cameroon area since the transition from a 2-dose to 3-dose IPTp-SP policy in Cameroon in 2012. Given that the effectiveness of this strategy is being challenged by rising levels of parasite resistance, this study provide evidence on the extent at which ≥ 3 doses of SP compared with the 2-dose IPTp-SP regimen is effective against PM infection and LBW in the study area.

This study revealed an increased in the percentage of women receiving the recommended three or more doses of IPTp in the area consistent with World Malaria Report 2016 [38]. About two (47.0%) out of five women received the full WHO-recommended schedule which is higher than 29.3% from the National Demographic Health Survey (DHS) data collected in 2011 in Cameroon [10]. An overall prevalence of 29.5% for three doses of IPTp-SP was obtained from Malaria Indicator Surveys (MIS) conducted in Burkina Faso, Ghana, Mali, Malawi, Kenya, Nigeria, Sierra Leone, and Uganda with considerable inter- and intra-country variations in the adequate uptake of IPTp [39].

Comparatively, the coverage of IPTp (≥ 3) in the Mount Cameroon area appears to be better than in many other countries in sub-Saharan Africa, except for Ghana where a higher coverage of 60% has been reported [40]. Ten years after implementation of IPTp- SP in Cameroon, coverage with at least two doses in this part of the country has increased from 64.7% in 2009 [34] to 81.7% in 2017 as stipulated by the WHO previous policy on IPTp-SP [41]. Most women who did not take chemoprophylaxis during their pregnancy were from semi-rural setting, however the coverage of the IPTp-SP doses and ITN usage were similar in both semi-rural and urban areas. This observation highlights the positive contribution of malaria control programmes towards bridging disparities and inequities in accessing malaria health services between rural and urban women [10, 42]. Access to chemoprophylaxis and ITNs among pregnant women have become more equitable in Cameroon due to provision of IPTp-SP free of charge and affordable essential drug schemes which lower costs [10].

In conformity with reports of several studies, ANC initiation in the first trimester or earlier in the second trimester as well as frequent ANC visits made before delivery allowed for more SP doses to be taken [34, 43, 44]. Uptake of ≥ 3 doses was also associated with type of health care facility where MDH had the highest coverage (72%). In Cameroon, the health district is the implementer unit of the health system and thus it not uncommon for the availability of SP at MDH to form the basis for high IPTp-SP coverage among pregnant women attending ANC in this facility. The relatively low uptake of SP at the level of the health centres may be due to unavailability of SP as a result of stock outs. During stock outs, IPTp is suspended until stocks are replenished or women are asked to buy SP from local drug vendors. Stock outs may be related to drug shortage at the central unit or challenges in supply chain management programs [45]. Periodic shortage of SP in rural health facilities has been reported as a major barrier to the implementation of IPTp-SP programme in some malaria endemic countries [46, 47].

Earlier studies of IPTp in Cameroon demonstrated a clear reduction in placental parasitaemia following receipt of SP [32, 48]. Also, data from a meta-analysis showed that ≥ 3 doses was associated with less placental malaria [8]. Contrarily, our study did not show prophylactic effectiveness of IPTp-SP in reducing PM infection. The risk of placental parasitaemia increased by 42% among delivery women having taken ≥ 3 doses. Although, PM parasitaemia was generally scarce as reported in previous studies [32], increase parasite load was noted mainly among women from urban areas who had adequate SP doses. The ineffectiveness of IPTp-SP on PM infection was noteworthy among primiparae. It is well known that in areas of intense transmission, primigravidae have higher vulnerability to malaria infection and its adverse consequences [1]. Thus, the effect of IPTp-SP against malaria should be more profound in these individuals than in secundi- and multigravidae as suggested by Valea et al. [49]. The lack of prophylactic effect of IPTp-SP in the prevention of malaria parasitaemia observed may be due to the presence of highly drug resistant *P. falciparum* parasites among infected pregnant women in south-western Cameroon. Agbor and Apinjoh [22] provide evidence of a high prevalence (51.1%) of *pfdhps* A581G mutation among pregnant women along the slope of mount Cameroon. Also, Chauvin et al. [21] reported *pfdhfr/pfdhps* octuple mutant alleles with A581G and A613S mutations among parasites isolated from pregnant women with symptomatic *P. falciparum* infection in Yaoundé, Cameroon. Although *pfdhps* K540E mutation was not observed in the study area [22], its presence, though rare, was confirmed in studies in Yaoundé [21]. In several countries in East Africa, SP resistance linked to vast predominance of highly resistant strains of *pfdhps* 540E and *pfdhps* 581G mutations in these populations has been associated with in vivo therapeutic failure to SP [11, 14–18, 50, 51]. IPTp use in areas with 581G mutation facilitate increase in parasite density whereby these highly resistant parasites out-compete the wild type populations and overgrow under drug pressure [14]. This may partly explain the increase placenta parasite load seen among women especially those from high IPTp-SP coverage areas, such as Muyuka. In line with reports from Benin by Moussiliou et al. [52], which highlight the inability of SP to ensure optimal antiplasmodial protection in late pregnancy, these present findings further suggest that SP is potentially failing in Central-West Africa. Future studies in the mount Cameroon area to link SP drug resistance molecular markers and efficacy of IPTp-SP are crucial taking into consideration the WHO recommendation to discontinue IPTp-SP in areas with A581G mutation > 10% [53].

Although ineffective in reducing PM infection, IPTp-SP showed improvement of infant outcomes in the area. Chemoprophylaxis with IPTp –SP (≥ 3 doses) was associated with reduced odds of LBW by 22%. From this study, the association between timing of ANC initiation and dosage frequency suggests that earlier dosing reduces odds of PM infection and thus improves birth weight outcome than late dosing which is linked to late antenatal enrolment. Comparable with reports from Burkina Faso [49], increased number of doses from 2 to ≥ 3 doses did not confer additional protection from LBW nor significantly increase birth weight. Gutman et al. [50] suggested that under conditions of resistance, more doses would be required to achieve the same benefit seen with only 2 doses in areas without resistance. SP resistance increases the minimum inhibitory concentration, leading to a shorter duration of prophylaxis. Interestingly, particularly among primiparae, adequate doses of SP was seen associated with improved birth weight outcome despite evidence of no parasitological response in clearance of placental infection. This is consistent with extensive reports on beneficial associations between the use of IPTp-SP and low birthweight still seen in areas where the efficacy of SP to clear parasitaemia has clearly decreased [11, 12]. Besides its anti-malarial activity, IPTp-SP appears to confer benefit through yet undefined pathways [19, 20, 54]. Sulfadoxine is a broad-spectrum antibiotic that possibly exerts an inhibitory effect against non-malaria causes of LBW and preterm birth [55]. Its antibacterial activity can lead to maternal and infant weight gain through changes in gut and vagina bacteria flora associated with indirect metabolic benefits and reduction in the impact of urogenital tract infections [56, 57], respectively. SP exposure inhibits (immunomodulatory role) maternal inflammatory responses to infections that are known to trigger preterm delivery [58].

### Limitations

This study has some limitations. These data were collected from a cross-sectional delivery survey. Although the effects of potential confounders have been corrected by using appropriate statistical methods, as in any observational study, unmeasured confounders, such as socioeconomic data may have had reasonable influence on the observations reported. Although SP was given as directly observed treatment (DOT) to ensure that pregnant women take the full dose, assessment of the reliability of IPTp-SP uptake is by evaluating the presence of measurable sulfa in maternal plasma during pregnancy. HIV screening is routinely undertaken during antenatal clinic visit, but the HIV status was not available for all study participants enrolled. However, the overall prevalence of HIV infection in the present study area was 4.6%. Despite these limitations, the data presented provide useful information on the coverage and effectiveness of IPTp-SP in the context increasing SP resistance parasite strains in the Mount Cameroon area.

## Conclusions

The current study has revealed a significant improvement in the uptake of ≥ 3 doses SP in the Mount Cameroon area with a coverage of 47.0%, which comparatively is better than that reported in many malaria-endemic regions in sub-Saharan Africa. Frequent visits to the ANC clinic, early uptake of the first dose of SP and type of health facility determined uptake of adequate doses of SP. The increasing trend in IPTp-SP uptake at ANCs in both rural and urban settings buttresses the positive contribution of malaria control programmes towards increasing uptake of health services and reducing disparities between rural and urban women. Prophylactic ineffectiveness of IPTp-SP in preventing placental malaria was observed in the study area. Though IPTp-SP still have beneficial effect in reducing odds of LBW as well as increase infant birth weight and maternal haemoglobin levels, no additional benefit was observed by adding a third dose of SP to the standard 2-dose regimen. These findings suggest SP is potentially failing in Central-West Africa and, therefore, future studies to assess the impact of *P. falciparum dhfr/dhps* resistant gene mutations on the efficacy of IPTp-SP in the Mount Cameroon area is critical.

## Supplementary information

**Additional file 1.** Maternal and infant outcomes by number of SP doses and parity. This file shows the occurrence of PM infection, anaemia, LBW as well as GMPMD, mean maternal Hb levels and birth weight in the different groups of SP doses received (≤ 1, 2, ≥ 3) among primiparae, secundiparae and multiparae women in the Mount Cameroon area.

**Additional file 2.** Comparison of crude and adjusted odd ratios of potential confounders associated with risk of placental malaria infection among parturient women in the Mount Cameroon area. This file shows percentage change in crude odd ratios after adjusting for possible confounders associated with risk of PM infection among parturient women in the Mount Cameroon area.

**Additional file 3.** Maternal and infant outcomes by number of SP doses and setting. This file shows the occurrence of PM infection, anaemia, LBW as well as GMPMD, mean maternal Hb levels and birth weight in the different groups of SP doses received (≤ 1, 2, ≥ 3) between women living in semi-rural and semi-urban setting in the Mount Cameroon area.

**Additional file 4.** Comparison of crude and adjusted odd ratios of potential confounders associated with risk of low birth weight in newborns of parturient women in the Mount Cameroon area. This file shows percentage change in crude odd ratios after adjusting for possible confounders associated with risk of LBW among parturient women in the Mount Cameroon area.

## Data Availability

All datasets generated and analyzed during the study are presented in the paper and its additional files.

## Abbreviations

IPTp-SP: Intermittent preventive treatment in pregnancy with sulfadoxine–pyrimethamine
PM: Placenta malaria
LBW: Low birth weight
ANC: Antenatal clinic
ITN: Insecticide-treated net
MMC: Mutengene medical centre
BHC: Bolifamba health centre
MHC: Munyenge integrated health centre
MDH: Muyuka district hospital
