# Supplementary material for: Coverage and effectiveness of intermittent preventive treatment in pregnancy with sulfadoxine–pyrimethamine (IPTp-SP) on adverse pregnancy outcomes in the Mount Cameroon area, South West Cameroon
Source: Malar J. 2020 Mar 2;19:100. doi: 10.1186/s12936-020-03155-2 (PMC7053117; doi:10.1186/s12936-020-03155-2)
Supplement: Supplementary file 1 — Additional file 1. Maternal and infant outcomes by number of SP doses and parity. This file shows the occurrence of PM infection, anaemia, LBW as well as GMPMD, mean maternal Hb levels and birth weight in the different groups of SP doses received (≤ 1, 2, ≥ 3) among primiparae, secundiparae and multiparae women in the Mount Cameroon area. [file 12936_2020_3155_MOESM1_ESM.docx]

**Additional table 1: Maternal and infant outcomes by number of SP doses and parity**

| Outcomes/  IPTp-SP  doses | Primiparae | | | Secundiparae | | | Multiparae | | |
| --- | --- | --- | --- | --- | --- | --- | --- | --- | --- |
|  | **≤ 1** | **2** | **≥ 3** | **≤ 1** | **2** | **≥ 3** | **≤ 1** | **2** | **≥ 3** |
| ^d^PM  % (n) | 9.7(3) | 19.1 (9) | 36.1 (22) | 17.4(4) | 24.4 (11) | 17.2(11) | 15.5 (9) | 10.3 (6) | 14.1 (11) |
| ^e^Anaemia % (n) | 76.9 (20) | 64.3 (27) | 67.3(37) | 63.6(14) | 52.4(22) | 60.7 (34) | 73.5 (36) | 71.4 (40) | 51.5 (35) |
| ^f^LBW  %(n) | 16.0 (4) | 7.1 (3) | 5.5 (3) | 13.6(3) | 4.8 (2) | 3.6 (2) | 12.6 (6) | 1.8 (1) | 4.3 (3) |
| ^g^GMPMD  (%)  (range) (n) | 5.8  (0.4 – 63.4)  (3) | 2.9  (0.2 – 68) (9) | 1.4  (0.1 -100) (21) | 0.6  (0.1 - 4.5) (4) | 0.2  (0.1 – 2.3) (10) | 1.1  (0.1 -99.4) (10) | 1.1  (0.1 – 63.4) (9) | 0.9  (0.1 – 68) (6) | 1.3  (0.1 – 100) (11) |
| ^h^Mean  Hb levels (g/dl) | 10.0 ± 1.4 | 10.4± 1.3 | 10.5 ± 1.5 | 10.1±1.5 | 10.6±1.6 | 10.5 ± 1.1 | 10.1±1.5 | 10.5 ±1.0 | 10.6 ± 1.7 |
| ^i^Mean  BWT (kg) | 3.1 ± 0.6 | 3.1± 0.5 | 3.3 ± 0.5 | 3.0 ± 0.6 | 3.4 ± 0.6 | 3.4 ± 0.6 | 3.1 ± 0.6 | 3.4 ± 0.5 | 3.4 ± 0.6 |

^d =^ PM significantly different between SP doses in primparae (p = 0.012) not secundiparae (p = 0.615) nor Multiparae (p = 0.696)

^e =^ Anaemia significantly different between SP doses in multiparae (p = 0.019) not primparae (p = 0.540) nor secundiparae (p = 0.607),

^f =^ LBW: counts are few for analysis.

^g^ = GMPMD not significantly different between SP doses in the different parity groups (p = 0.281)

^h^ = Mean Hb levels not significantly different between SP doses in the different parity groups ( p = 0.901)

^i^ = Mean BWT significantly different between SP doses in the different parity groups (p = 0.028)

P - values are from Pearson Chi-square test (categorical variables) and ANOVA (continuous variables)
