# Supplementary material for: Coverage and effectiveness of intermittent preventive treatment in pregnancy with sulfadoxine–pyrimethamine (IPTp-SP) on adverse pregnancy outcomes in the Mount Cameroon area, South West Cameroon
Source: Malar J. 2020 Mar 2;19:100. doi: 10.1186/s12936-020-03155-2 (PMC7053117; doi:10.1186/s12936-020-03155-2)
Supplement: Supplementary file 2 — Additional file 2. Comparison of crude and adjusted odd ratios of potential confounders associated with risk of placental malaria infection among parturient women in the Mount Cameroon area. This file shows percentage change in crude odd ratios after adjusting for possible confounders associated with risk of PM infection among parturient women in the Mount Cameroon area. [file 12936_2020_3155_MOESM2_ESM.docx]

**Additional file 2: Comparison of crude and adjusted odd ratios of potential confounders associated with risk of placental**

**malaria infection among parturient women in the mount Cameroon area**

| Variable | Confounding variables | ^#^Crude odds ratio  (95% CI) | *P-value | ^$^Adjusted  odds ratio  (95% CI) | P-value | % change in crude odds ratio |
| --- | --- | --- | --- | --- | --- | --- |
| Setting | Semi-rural | 1.56 (0.99 – 2.56) | 0.056 | 1.85 (1.12 – 3.04) | 0.016 | 20 |
| Parity | Primiparity | 2.09 (1.19 – 3.68) | 0.034 | 2.13 (1.19 – 3.81) | 0.011 | 2 |
| IPTp-SP  Dosage frequency | ≥3 SP dose | 1.66 (0.89 – 3.10) | 0.245 | 2.36 (1.41 – 4.87) | 0.021 | 42 |
| Trimester of first ANC | Second trimester | 0.60 (0.34 – 1.05) | 0.085 | 0.39 (0.20 – 0.74) | 0.004 | 35 |

^#^ values calculated using confidence interval calculator * values from Pearson Chi-square test for homogeneity, ^$^ values from multinominal regression analysis, ANC= Antenatal clinic, IPTp-SP = intermittent preventive treatment in pregnancy with sulphadoxine-pyrimethamine, CI = confidence interval
