# Supplementary material for: Coverage and effectiveness of intermittent preventive treatment in pregnancy with sulfadoxine–pyrimethamine (IPTp-SP) on adverse pregnancy outcomes in the Mount Cameroon area, South West Cameroon
Source: Malar J. 2020 Mar 2;19:100. doi: 10.1186/s12936-020-03155-2 (PMC7053117; doi:10.1186/s12936-020-03155-2)
Supplement: Supplementary file 3 — Additional file 3. Maternal and infant outcomes by number of SP doses and setting. This file shows the occurrence of PM infection, anaemia, LBW as well as GMPMD, mean maternal Hb levels and birth weight in the different groups of SP doses received (≤ 1, 2, ≥ 3) between women living in semi-rural and semi-urban setting in the Mount Cameroon area. [file 12936_2020_3155_MOESM3_ESM.docx]

**Additional file 3: Maternal and infant outcomes by number of SP doses and setting**

| Outcome/  IPTp-SP doses | semi -rural | | | semi-urban | | | |
| --- | --- | --- | --- | --- | --- | --- | --- |
|  | **≤ 1** | **2** | **≥ 3** | **≤ 1** | **2** | **≥ 3** |  |
| ^j^ PM %(n) | 16.4(11) | 20.3 (15) | 26.8 (26) | 11.1(5) | 14.5 (11) | 17.0 (18) |  |
| ^k^Anaemia % (n) | 86.5 (45) | 75.0 (48) | 69.9 (51) | 55.6 (25) | 53.9(41) | 51,9 (55) |  |
| ^l^LBW %(n) | 23.1(12) | 7.8 (5) | 8.1 (6) | 2.3 (1) | 1.3(1) | 1.9 (2) |  |
| ^g^GMPMD % (range) (n) | 1.5  (0.2 – 7.5) (11) | 0.7  (0.1 – 33) (15) | 0.6  (0.1 -16.0) (24)  - | 0.6  (0.1 – 63.4) (5) | 1.2  (0.1 – 68) (10) | 3.4  (0.1- 100) (18) |  |
| ^h^Mean Hb levels (g/dl) | 9.7 ± 1.3 | 10.0 ±1.4 | 10.0 ± 1.8  - | 10.5 ± 1.5 | 10.9 ± 1.0 | 10.9 ± 1.0 |  |
| ^i^Mean BWT (kg) | 3.0 ± 0.7 | 3.3 ± 0.6 | 3.3 ± 0.6  - | 3.2 ± 0.4 | 3.3 ± 0.4 | 3.4 ± 0.5 |  |

P- values are obtained from Pearson Chi-square test (categorical variables) and ANOVA (continuous variables);

^j =^ PM not significantly different between SP doses in different setting; semi-rural (p = 0.264), semi-urban (p = 0.645)

^k =^ Anaemia not significantly different between SP doses in different setting; semi-rural (p = 0.094), semi-urban (p = 0.909)

^l =^ LBW significantly different between SP doses in rural setting (p = 0.017), semi-urban (counts are few for analysis)

^m =^ GMPMD significantly different between SP doses in different settings (p < 0.001)

^n^ = Mean Hb levels significantly different between SP doses in different settings (p < 0.001)

^o^ = Mean BWT not significantly different between SP doses in different settings (p = 0.203)
