# Supplementary material for: Coverage and effectiveness of intermittent preventive treatment in pregnancy with sulfadoxine–pyrimethamine (IPTp-SP) on adverse pregnancy outcomes in the Mount Cameroon area, South West Cameroon
Source: Malar J. 2020 Mar 2;19:100. doi: 10.1186/s12936-020-03155-2 (PMC7053117; doi:10.1186/s12936-020-03155-2)
Supplement: Supplementary file 4 — Additional file 4. Comparison of crude and adjusted odd ratios of potential confounders associated with risk of low birth weight in newborns of parturient women in the Mount Cameroon area. This file shows percentage change in crude odd ratios after adjusting for possible confounders associated with risk of LBW among parturient women in the Mount Cameroon area. [file 12936_2020_3155_MOESM4_ESM.docx]

| Variable | Confounding variable | ^#^Crude odds ratio  (95% CI) | *P-value | ^$^Adjusted  odds ratio  (95% CI) | P-value | % change in crude odd ratio |
| --- | --- | --- | --- | --- | --- | --- |
| Setting | Semi-rural | 7.62(2.59 – 22.52) | <0.001 | 5.29 (1.35 – 16.15) | 0.003 | 31 |
| PM infection | Positive | 2.45 (1.05 – 5.68) | 0.032 | 2.33 (0.90 – 6.07) | 0.083 | 0 |
| Anaemia  status | Anaemic | 7.71 (1.8 - 33.02) | 0.001 | 4.60 (1.03 – 20.57) | 0.046 | 40 |
| IPTp-SP dosage frequency | ≥3 SP doses | 0.40 (0.16 – 1.0) | 0.006 | 0.31 (0.11 – 0.87) | 0.027 | 22 |
|  | 2 doses | 0.29 (0.11 – 0.79) | 0.006 | 0.32 (0.11 – 0.93) | 0.036 | 10 |

**Additional file 4:** **Comparison of crude and adjusted odd ratios of potential confounders associated with risk of low birth weight in newborns of parturient women in the mount Cameroon area**

^#^ values calculated using confidence interval calculator,* values from Pearson Chi-square test for homogeneity, **^$^** values from multinominal regression analysis ANC= Antenatal clinic, IPTp-SP = intermittent preventive treatment in pregnancy with sulphadoxine pyrimethamine, PM: placental malaria, CI = confidence interval
